# Sentinel Lymph Node Mapping in Endometrial Cancer in the Molecular Era: Current Evidence, Clinical Controversies, and Unresolved Questions

**DOI:** 10.3390/jcm15218690

**Published:** 2026-11-07

**Authors:** Raffaele Tinelli, Felice Sorrentino, Daniela Surico, Alessandro Messina, Stefano Angioni, Giorgio Bogani, Carmen Imma Aquino, Michele Peiretti, Alessandro Libretti

**Affiliations:** 1 Department of Obstetrics and Gynecology, “Valle d’Itria” Hospital, 74015 Martina Franca, Italy; raffaeletinelli@gmail.com; 2 Department of Medical and Surgical Sciences, Institute of Obstetrics and Gynecology, University of Foggia, 71122 Foggia, Italy; 3 Department of Gynaecology and Obstetrics, University Hospital Maggiore Della Carità, Corso Mazzini, 18, 28100 Novara, Italy; daniela.surico@med.uniupo.it (D.S.); c.immaquino@gmail.com (C.I.A.);; 4 Department of Obstetrics and Gynecology, University Hospital “Degli Infermi”, 13875 Ponderano, Italy; alessandro.messina@aslbi.piemonte.it; 5 Division of Gynecology and Obstetrics, Department of Surgical Sciences, University of Cagliari, 09124 Cagliari, Italy; sangioni@aoucagliari.it (S.A.); mpeiretti@aoucagliari.it (M.P.); 6 Gynecologic Oncologic Unit, Fondazione IRCCS Istituto Nazionale dei Tumori di Milano, 20133 Milan, Italy; giorgiobogani@yahoo.it

**Keywords:** endometrial cancer, sentinel lymph node, indocyanine green, molecular classification, ultrastaging, failed mapping, para-aortic lymph nodes, high-risk histology

## Abstract

Sentinel lymph node (SLN) mapping is an established approach to nodal staging in presumed uterine-confined endometrial cancer, but its integration with molecular risk assessment raises unresolved clinical questions. This narrative review examines tracer and injection-site selection, persistent mapping failure, high-risk histologies, para-aortic assessment, low-volume nodal disease, and interpretation across POLEmut, MMRd, NSMP and p53abn tumors. A focused literature update and bibliographic verification were performed in October 2026. Cervical indocyanine green injection with near-infrared imaging supports reproducible pelvic mapping; comparative tracer studies favor it over blue dye. Prospective high-risk cohorts demonstrate high diagnostic accuracy when a complete algorithm is applied, but do not establish survival non-inferiority within every histologic or molecular subgroup. An unmapped hemipelvis requires technical reassessment and management according to baseline staging indication and operative feasibility. Negative pelvic SLNs do not exclude all para-aortic disease; additional assessment should address a defined treatment-planning question. Micrometastases constitute nodal metastasis, whereas isolated tumor cells are reported as pN0(i+). Molecularly annotated cohorts refine prognostic interpretation, but do not validate subgroup-specific treatment rules for low-volume disease. Future trials should evaluate treatment changes, morbidity and oncologic outcomes, rather than detection alone. The review distinguishes current staging practice from prognostic observations and hypotheses requiring prospective validation.

## 1. Introduction

Nodal status contributes to anatomic staging, prognosis and adjuvant treatment planning in endometrial cancer. Systematic lymphadenectomy provides staging information at the cost of additional operative burden and lymphatic morbidity. Randomized trials of systematic pelvic lymphadenectomy did not demonstrate a survival advantage in the early-stage populations studied [1,2]; these results do not independently settle the role of para-aortic assessment or every contemporary high-risk subgroup. SLN mapping aims to preserve clinically useful staging information while reducing the extent of dissection [3]. Its value depends on the complete surgical algorithm and appropriate pathological assessment, rather than fluorescence alone.

Prospective studies addressed different questions. FIRES and SENTI-ENDO evaluated diagnostic accuracy; FILM compared tracer detection; and SHREC and SENTOR examined algorithm-based staging in high-risk or high-grade cohorts [4,5,6,7,8]. These designs should not be conflated with randomized comparisons of recurrence or survival. Contemporary guidance incorporates SLN mapping in presumed uterine-confined disease [9], while molecular classification and FIGO 2023 provide additional context for interpreting nodal findings [10].

Recent reviews already summarize technique, indications, pathology and molecular integration [11,12,13,14,15,16,17]. The remaining clinical problem is how to use this evidence when mapping is incomplete, histology is aggressive, para-aortic risk remains uncertain, or ultrastaging reveals a small tumor deposit. Molecularly annotated cohorts can identify associations between tumor biology and nodal findings [18,19,20,21,22], but changing the extent of surgery or adjuvant treatment requires evidence beyond association.

We therefore examine decisions in which the evidence may change management: selection of tracer and injection site, rescue after unilateral or bilateral mapping failure, staging in high-risk disease, interpretation of low-volume deposits, and selective para-aortic assessment. Non-standard settings are discussed separately because evidence from primary staging cannot automatically be generalized to post-treatment or reoperative surgery. Throughout, diagnostic validation, prognostic information and demonstrated treatment benefit are considered as distinct endpoints.

## 2. Methods

This is a focused narrative review, not a systematic review or meta-analysis. The literature search covered the period from 1 January 2000 to 22 October 2026. Searches of MEDLINE-indexed records through Europe PMC were performed using the following query: (TITLE_ABS: "endometrial cancer" OR TITLE_ABS: "endometrial carcinoma") AND (TITLE_ABS: "sentinel lymph node" OR TITLE_ABS: "sentinel node") AND FIRST_PDATE: [2000-01-01 TO 2026-10-22].A supplementary search added (TITLE_ABS: neoadjuvant OR TITLE_ABS: reoperative OR TITLE_ABS: restaging) to the same disease/mapping expression, using the same publication date range. The cited primary studies and the 2025 ESGO–ESTRO–ESP guidance were also verified. Historical references were checked individually by PMID, DOI, or title.

Records were selected purposively for direct relevance to the clinical questions: prospective diagnostic validation, comparative outcomes, molecularly annotated nodal cohorts, pathological assessment, mapping rescue, and current guidance. Recent reviews were examined to assess overlap and contextualize the contribution of this review. Preprints were not used to support practice recommendations. Trial protocols were distinguished from completed outcome studies. No duplicate screening, formal risk-of-bias assessment, pooled analysis, or exhaustive accounting of all potentially eligible publications was undertaken. The focused update is reproducible at the query level, but selection remains interpretive and subject to bias. Comparisons are limited by variation in population, surgeon experience, algorithm, ultrastaging and adjuvant treatment. No original patient data were collected.

## 3. What Is New Beyond Recent Reviews?

The purpose of this article is to connect unresolved clinical decisions with the level of evidence available to answer them. Earlier reviews cover the principal technical and clinical domains [11,12,13,14,15]. Bogani et al. [16] specifically address molecular integration, and Glaser and Ostby [17] discuss contemporary practice and the addition of molecular profiling. Table 1 therefore acknowledges overlapping coverage. Our contribution is to distinguish an actionable staging recommendation from a prognostic observation or a hypothesis for treatment modification at each decision point.

The 2025 prognostic cohort [18] belongs to the established molecular evidence base, rather than constituting an advance introduced here. The 2026 Scarpelli and Pellicer cohorts [20,21] and PROMISE-EC [22] extend this evidence. PROMISE-EC included 2003 patients in a retrospective multicenter analysis; macrometastatic SLN involvement retained independent prognostic information, whereas the association of lower-volume burden with recurrence attenuated after adjustment. These findings support integrating nodal and uterine/molecular information, but cannot identify a causal benefit from a particular treatment. The SENTIMETREP53 protocol [23] illustrates the next evidentiary step: testing survival after a molecularly defined surgical strategy prospectively. Its design is relevant; outcome superiority or non-inferiority cannot yet be inferred from the protocol.

## 4. Technical Foundations That Still Matter

### 4.1. Tracer Selection: ICG, Blue Dyes, and Technetium-99m

The tracer influences mapping success, workflow, cost, and the anatomical information available to the surgeon. Indocyanine green (ICG) visualized with near-infrared imaging has become the preferred tracer because it provides high overall and bilateral detection, rapid intraoperative visualization, and avoids preoperative nuclear-medicine logistics. In a prospective comparison of 100 patients, How et al. found that ICG had higher overall and bilateral detection than blue dye (87% vs. 71% overall; 65% vs. 43% bilateral), whereas performance was similar to technetium-99m for overall and bilateral detection [24]. Comparative studies likewise reported higher bilateral mapping with ICG than conventional radiocolloid/blue dye strategies [25,26].

Blue dye is a visual tracer that requires no near-infrared platform, but lower bilateral detection, tissue staining and hypersensitivity limit its use. In a randomized comparison, adding blue dye to ICG plus technetium did not improve detection [27]. Technetium-99m supports gamma-probe localization and preoperative lymphoscintigraphy or SPECT/CT, but requires nuclear-medicine coordination and radiotracer handling. ICG avoids that workflow but requires near-infrared equipment. A history of an iodinated-contrast reaction should be distinguished from a documented ICG reaction; the risk assessment should consider the specific agent and reaction, formulation and local perioperative protocol [28].

**Figure 1:**
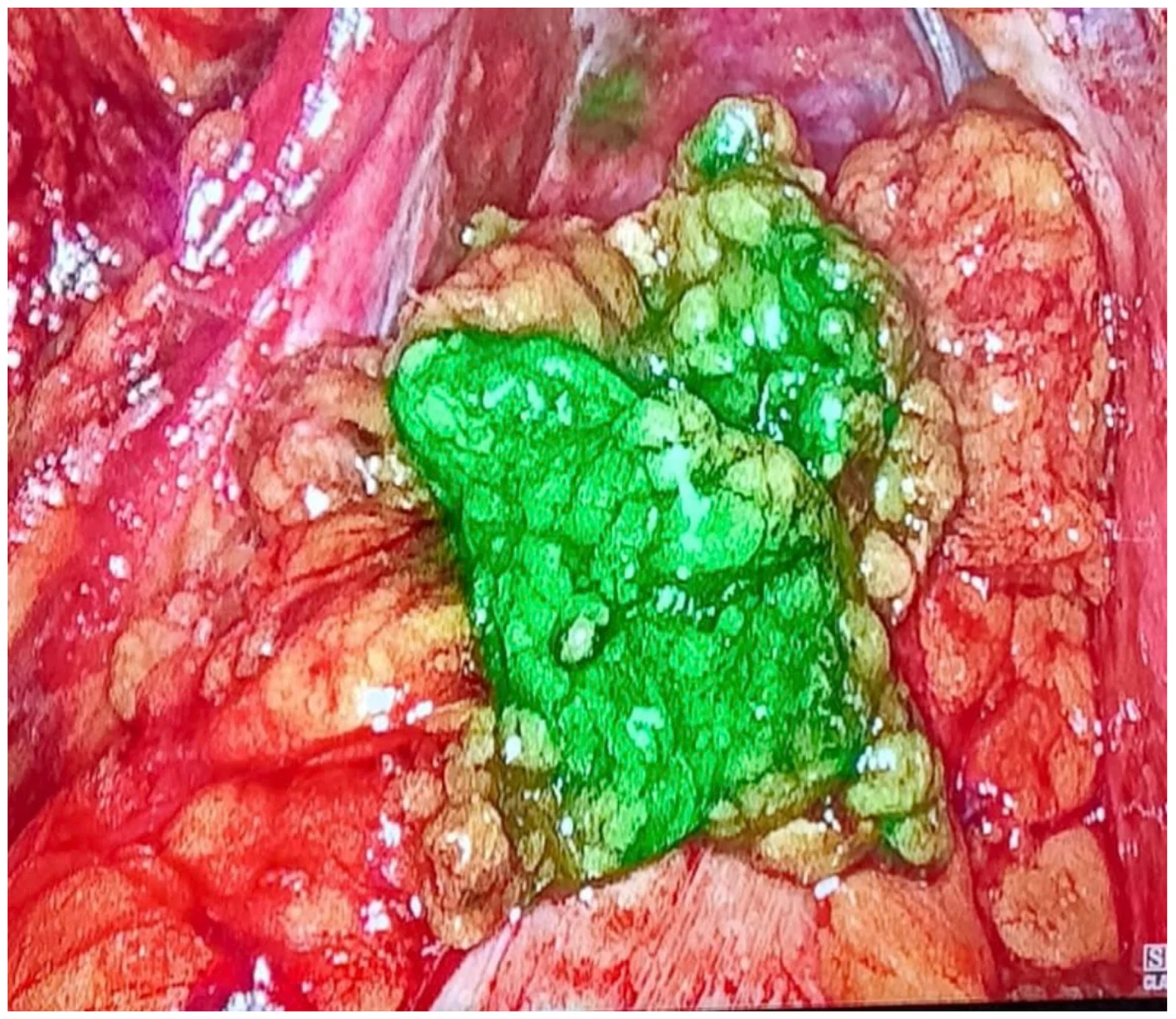
Intraoperative near-infrared fluorescence imaging during sentinel lymph node mapping with indocyanine green (ICG), showing fluorescent lymphatic tissue and mapped sentinel nodal tissue during minimally invasive staging of endometrial cancer. Original image provided by the author.

The practical preference for ICG does not imply universal superiority over every radiocolloid-based protocol. The How comparison found similar detection with ICG and technetium [24], while the multicenter Papadia cohort reported higher bilateral detection with ICG than technetium plus blue dye [26]. Detection depends on technique, population and experience. Superparamagnetic iron oxide remains an investigational alternative supported by early feasibility data [29], rather than an established replacement. Figure 1 illustrates intraoperative ICG fluorescence imaging during SLN mapping. The practical advantages, limitations, and current clinical roles of the available tracers are summarized in Table 2.

**Table 2:** Practical comparison of tracers for SLN mapping in endometrial cancer.

Tracer	Strengths	Limitations	Evidence Signal	Current Role
ICG + NIR	High bilateral detection; real-time visualization; no nuclear medicine	Requires NIR platform; fluorescence can diffuse if injection/dissection timing is suboptimal	Higher detection than blue dye; similar or better bilateral detection than conventional combinations [24,25,26]	Preferred tracer in most contemporary algorithms
Technetium-99m	Good detection; preoperative lymphoscintigraphy/SPECT possible	Nuclear-medicine logistics; radiation handling; additional preoperative workflow	Comparable overall detection to ICG in direct prospective comparison [24]	Valid alternative or adjunct where infrastructure exists
Blue dye	Low cost; simple visual identification	Lower bilateral detection; tissue staining; hypersensitivity risk	Inferior to ICG in comparative studies [24]	Generally not preferred as a sole tracer
ICG + Tc-99m ± blue	Potential redundancy and multimodal confirmation	More complex and costly; blue dye adds little to ICG + Tc-99m	No meaningful gain from adding blue dye to ICG + Tc-99m in a randomized comparison [27]	Selected centers/research

### 4.2. Injection Site and Uterine Lymphatic Drainage

The injection site determines which uterine lymphatic pathways are preferentially visualized. Cervical injection is technically simple, reproducible, and provides the highest and most consistent pelvic bilateral mapping. From the cervix, lymphatic drainage predominantly follows parametrial pathways toward obturator, external iliac, internal iliac, and common iliac nodal basins. This explains why cervical injection is particularly effective for pelvic staging.

The uterine corpus and fundus also have cranial drainage along the ovarian vessels toward para-aortic nodes. Injection nearer the endometrium or fundus may visualize this route more often, but a visible route is not a complete map of metastatic spread. In the randomized MITO comparison, 151 patients were analyzed: hysteroscopic injection showed numerically greater para-aortic detection, without a statistically significant difference in the primary para-aortic detection endpoint. Isolated para-aortic SLNs were identified in 5.8% with hysteroscopic injection and 0% with cervical injection; overall and pelvic detection favored cervical injection [30]. These results support an anatomical trade-off rather than proven clinical superiority of hysteroscopic injection.

**Figure 2:**
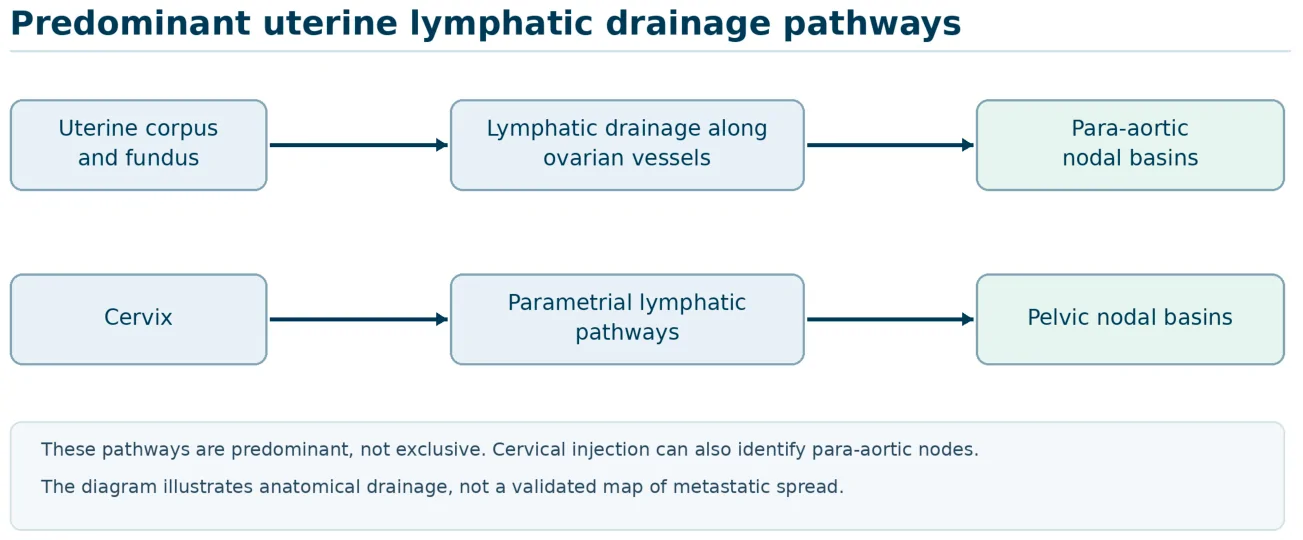
Original schematic of predominant uterine lymphatic drainage pathways. Drainage from the corpus and fundus may follow the ovarian vessels to para-aortic nodal basins, whereas cervical injection preferentially demonstrates parametrial routes to pelvic nodal basins. These pathways are predominant rather than exclusive; cervical injection may also identify para-aortic nodes. The schematic does not represent a validated map of metastatic spread.

Current evidence supports cervical injection as the standard approach because the clinical objective is reliable bilateral pelvic staging with a validated algorithm. Fundal, subserosal, or hysteroscopic approaches should be viewed as investigational or selective strategies rather than routine replacements. Their potential value may be greatest in studies designed specifically to answer whether additional direct para-aortic mapping changes management in high-risk molecular or histologic subgroups. Figure 2 summarizes the predominant uterine lymphatic drainage pathways. The advantages, limitations, and clinical implications of the different injection sites are summarized in Table 3.

**Table 3:** Injection-site trade-offs.

Injection Site	Dominant Mapping Pattern	Advantages	Limitations/Clinical Implication
Cervical (superficial + deep)	Pelvic: obturator/external/internal/common iliac	Simple; reproducible; best validated bilateral pelvic mapping	Lower direct para-aortic detection; current reference approach
Hysteroscopic/endometrial/peritumoral	Numerically greater direct para-aortic visualization; primary MITO comparison not statistically significant	Potentially mirrors tumor drainage more directly	Technically more complex; pelvic and overall detection favored cervical injection in MITO [30]
Fundal/subserosal/myometrial	May access ovarian-vessel/infundibulopelvic pathway	Potentially increases para-aortic mapping	Evidence limited and heterogeneous; should not replace validated cervical algorithm routinely

### 4.3. Pathologic Ultrastaging and Low-Volume Nodal Disease

Ultrastaging supplements routine hematoxylin-and-eosin examination with additional levels and, commonly, cytokeratin immunohistochemistry in initially negative SLNs. Protocols vary; Chiang et al. evaluated a defined two-level approach and a limited immunohistochemical protocol, illustrating the need to report the actual laboratory method [31]. Reports should specify basin/laterality, node counts, largest contiguous tumor deposit and extranodal extension. Macrometastases measure >2 mm; micrometastases measure >0.2–2 mm or exceed the applicable 200-cell threshold; ITCs are small deposits reported as pN0(i+) under the applicable staging criteria [10,31]. Size and cell-count rules should be applied consistently rather than classifying a focus from immunostaining alone.

Micrometastases constitute metastatic nodal involvement for staging; ITCs are reported as pN0(i+) and do not by themselves establish stage IIIC disease. A positive cytokeratin stain requires morphologic confirmation of tumor cells. Micrometastases warrant discussion within node-positive treatment pathways, whereas ITCs alone should not automatically trigger the same escalation. Uterine factors and molecular classification provide context, but no validated molecularly stratified treatment rule establishes the benefit of escalation or de-escalation specifically for ITCs or micrometastases [9,32,33,34]. Their biological and therapeutic significance within each molecular subgroup remains incompletely defined.

Observational data further caution against treating ITCs and micrometastases as a single category. A multicenter ITC cohort found associations with adverse uterine features, while molecular patterns differed from those of micro-/macrometastatic disease [35]. A 2025 meta-analysis reported associations between adjuvant treatment and improved outcomes in selected low-volume groups, but treatment selection and retrospective confounding limit causal inference [36]. These findings support multidisciplinary interpretation; they do not establish a molecularly specific benefit from escalation.

## 5. Unilateral and Failed Mapping: A Side-Specific Problem

Failed mapping should be defined by hemipelvis. Bilateral mapping is the desired endpoint; unilateral detection means that one hemipelvis is successfully staged and the contralateral hemipelvis remains unmapped. This distinction is clinically important because a statement such as “systematic lymphadenectomy remains indicated when SLNs are not identified” is too imprecise and may imply unnecessary bilateral dissection.

Remove mapped SLNs and all grossly suspicious nodes regardless of fluorescence. Assess mapping separately on each hemipelvis. For an unmapped side, reassess exposure, tracer delivery and visible lymphatic channels; consider cervical ICG reinjection before declaring persistent failure [37]. The next decision is whether nodal staging remains indicated for that patient, rather than whether unilateral fluorescence alone mandates dissection. In high–intermediate/high-risk disease, persistent failure supports side-specific pelvic lymphadenectomy under the applicable staging algorithm [9,38,39,40]. Bilateral failure requires the same assessment on both sides.

**Figure 3:**
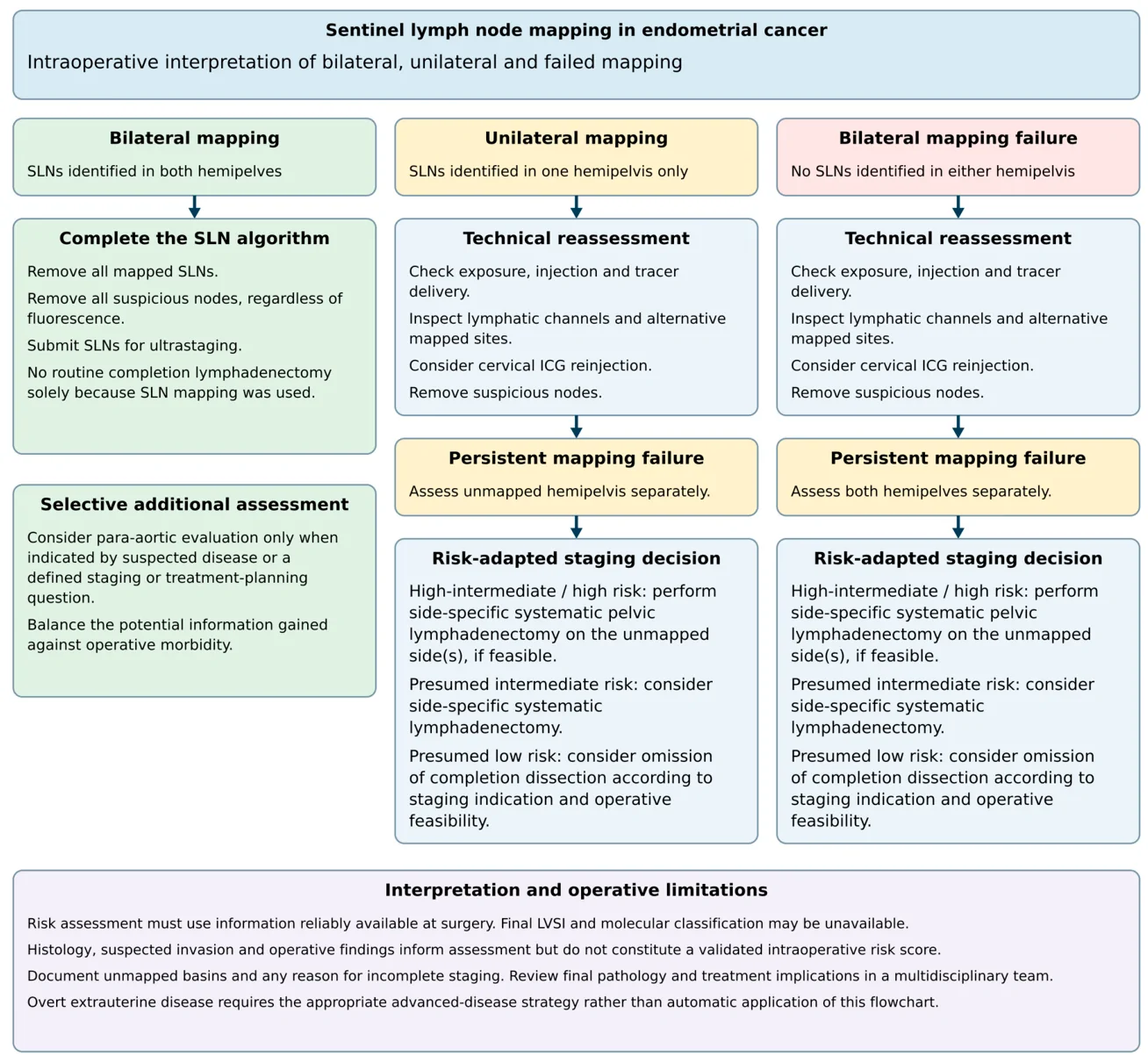
Risk-adapted intraoperative management according to sentinel lymph node mapping results. The flowchart distinguishes bilateral mapping, unilateral mapping, and bilateral mapping failure. Management of persistent failure follows the baseline staging indication, presumed risk category and operative feasibility [9]. Risk assessment uses information reliably available at surgery; final LVSI and molecular classification may be unavailable. This is an interpretive framework, not a prospectively validated decision rule.

For patients judged low risk using information reliably available at surgery, automatic completion lymphadenectomy is not justified solely by mapping failure; omission or consideration of backup dissection should follow current guidance and individualized staging needs. High-grade histology, suspected deep invasion, suspicious nodes, or other adverse findings favor completing indicated staging. Final LVSI and molecular results may be unavailable intraoperatively and should not be presented as universally usable rescue criteria. POLEmut status alone does not validate omission of backup staging, and p53abn status alone does not prescribe its anatomic extent. If operative risk prevents completion, document the unmapped basin and review the implications after final pathology. Imaging may evaluate suspected extrauterine disease but cannot certify a microscopically negative unmapped hemipelvis. In presumed intermediate-risk disease, side-specific systematic lymphadenectomy may be considered after persistent mapping failure [9]. Figure 3 summarizes this risk-adapted approach; the displayed features are clinical prompts rather than a validated risk classification.

Predictors depend on whether failure is defined as absence of bilateral mapping or absence of any SLN. The Raffone meta-analysis associated failure with low injected ICG volume, advanced stage, enlarged nodes and nodal involvement [41]. A later prospective cohort identified higher BMI and age as independent predictors of bilateral failure [42]; these findings should not be treated as universal thresholds or proof that a particular patient cannot map. Multicenter cohorts provide additional context [43,44]. Suspicious non-fluorescent nodes still require assessment because tumor-related obstruction may disrupt drainage. Table 4 summarizes the main clinical scenarios, evidence-based approaches, and unresolved questions concerning sentinel lymph node mapping, including unilateral and bilateral mapping failure.

**Table 4:** Clinical scenarios, suggested interpretation, and evidence gaps.

Clinical Scenario	Evidence-Based Approach	Avoid	Key Unresolved Question
Bilateral pelvic mapping with ICG	Remove mapped SLNs and suspicious nodes; ultrastaging	Routine completion lymphadenectomy solely because SLN was used	Which molecular subgroups can safely undergo further de-escalation?
Unilateral mapping despite reinjection	Treat by hemipelvis; side-specific nodal assessment of unmapped side when staging is indicated, especially high-risk disease	Calling this complete mapping or performing automatic bilateral full dissection	Can molecular/preoperative models identify patients who can omit backup dissection?
No mapping	Reassess technique, remove suspicious nodes, consider reinjection; perform risk-appropriate nodal assessment	Assuming negative nodes because no fluorescence is seen	Best rescue algorithm and role of alternative tracers.
High-risk histology	SLN acceptable in experienced centers using validated algorithm; low threshold for side-specific completion after failure	Absolute statement that SLN always replaces lymphadenectomy	Which high-risk molecular phenotypes need para-aortic information?
ITCs versus micrometastases	Report ITCs as pN0(i+); micrometastases are node-positive. Review uterine and molecular risk without assuming a validated subtype-specific treatment rule	Treating every low-volume focus as biologically equivalent	Molecularly stratified adjuvant benefit.
Pelvic SLN macrometastasis	Individualize para-aortic evaluation and adjuvant planning	Uniform para-aortic dissection regardless of treatment consequence	When does para-aortic staging change fields or systemic therapy?
Post-neoadjuvant surgery	Use cautiously; acknowledge limited validation	Presenting SLN as validated standard after neoadjuvant therapy	Prospective detection and false-negative data.

## 6. Is SLN Mapping Adequate in High-Risk Histologies?

High-grade endometrioid carcinoma, serous carcinoma, clear-cell carcinoma, carcinosarcoma, deep myometrial invasion, and substantial LVSI increase concern for nodal and extrauterine disease. The evidence must be separated into detection performance, diagnostic accuracy, anatomic staging, treatment consequences, and recurrence or survival outcomes. Histologic categories and molecular subgroups overlap but are not interchangeable: p53abn is a molecular category, not a synonym for serous carcinoma.

SHREC prospectively evaluated 257 patients with presumed stage I–II high-risk disease. Bilateral detection was 95%; the ICG algorithm had 98% sensitivity and 99.5% negative predictive value, while the overall algorithm incorporating suspicious nodes reached 100% sensitivity. Two patients had isolated para-aortic metastases [5]. SENTOR included 156 patients, 126 with high-grade disease; patient-level detection was 97.4%, bilateral detection 77.6%, sensitivity 96%, and false-negative rate 4% [6]. These are diagnostic-validation results obtained with reference lymphadenectomy, not randomized survival comparisons. Table 5 separates these endpoints.

SLN-based staging is an accepted strategy in appropriately selected patients with presumed uterine-confined high-risk cancer when an experienced team applies a validated algorithm, removes suspicious nodes, and uses ultrastaging [9]. This does not mean mapped-node excision alone is adequate after unresolved mapping failure. Retrospective comparisons in serous/clear-cell carcinoma, carcinosarcoma, and mixed high-risk cohorts provide outcome data [45,46,47], but selection bias, differences in adjuvant therapy, and small histology-specific samples limit claims of equivalence. Absence of a statistically significant survival difference is not proof of non-inferiority. The cited ALICE publication describes a randomized trial protocol; it is not a report of mature survival results [48].

For serous and clear-cell carcinoma, the principal concerns include occult extrauterine spread, para-aortic involvement and the effect of staging on radiation planning. For carcinosarcoma, available SLN outcome comparisons are retrospective and should not be interpreted as definitive prospective validation. Molecularly defined p53abn disease remains anatomically staged even when adverse biology already supports intensive adjuvant consideration. Across these groups, diagnostic accuracy does not independently establish recurrence or survival equivalence, and pelvic SLN assessment does not replace other histology-appropriate staging procedures. No subtype-specific survival benefit from additional lymphadenectomy can be inferred from the diagnostic studies.

## 7. SLN Mapping in the Molecular Era

The molecular classification comprises POLE-mutated (POLEmut), mismatch repair-deficient (MMRd), no specific molecular profile (NSMP), and p53-abnormal (p53abn) tumors. Valid assignment uses established sequencing and immunohistochemical criteria; a POLE variant must not be assumed pathogenic merely because it is present. Molecular information complements uterine and anatomic findings in FIGO 2023 and contemporary risk stratification [9,10]. Histology and molecular subgroup remain related but distinct descriptors.

Molecularly annotated observational studies report differences in nodal involvement and prognosis [18,19,20,21]. In the 2025 cohort, macrometastatic SLNs provided independent prognostic information in MMRd disease; associations in other subgroups were less consistent [18]. These findings describe risk, not a treatment-by-subgroup interaction. Sparse events, subgroup size, stage distribution and adjuvant treatment can influence the apparent incremental value of nodal status. A nonsignificant association in one subgroup does not show that nodal assessment is dispensable.

A practical interpretation starts with three separate questions: what is the anatomic nodal stage; what additional prognostic context does molecular classification provide; and is there evidence that a specific treatment improves outcomes in this setting? POLEmut tumors generally have favorable prognosis, but this does not establish that staging can be omitted or that node-positive disease can routinely receive the same de-escalation as early-stage disease. MMRd status may add therapeutic information in defined disease settings, but its association with nodal outcomes does not prove benefit from a specific adjuvant regimen for low-volume metastases. NSMP is heterogeneous; grade, estrogen-receptor status and uterine factors remain relevant. In p53abn disease, adverse baseline prognosis does not remove the anatomic or radiation-planning value of nodal status [9,18,19,20,21]. Table 6 distinguishes these interpretations from unvalidated treatment inferences.

## 8. The Para-Aortic Dilemma

Isolated para-aortic metastases without pelvic nodal involvement are uncommon but clinically relevant. Cervical ICG injection primarily maps pelvic pathways and may not directly visualize the fundal lymphatic route along the ovarian vessels. This anatomical limitation has generated concern that a pelvic-only SLN strategy could miss isolated para-aortic disease. Ultrastaging of pelvic nodes can reduce the apparent prevalence of truly isolated para-aortic dissemination by identifying otherwise occult pelvic low-volume disease [49].

Para-aortic assessment should be considered in relation to a concrete management decision. Suspicious para-aortic nodes on imaging or at surgery, bulky or macrometastatic pelvic nodes, and combinations of high-grade histology, deep invasion or substantial LVSI raise concern [5,6,9,49]. These are reasons for individualized assessment, not a validated score mandating systematic para-aortic dissection. Negative pelvic SLNs reduce but do not eliminate para-aortic risk. High-risk histology or p53abn status with negative pelvic SLNs does not, by itself, establish a benefit from routine para-aortic dissection.

Cross-sectional imaging, with selective functional imaging when appropriate, may help evaluate suspected para-aortic or distant disease and guide biopsy or surgical assessment; negative imaging does not exclude microscopic metastases. Demonstrating para-aortic involvement changes the anatomic nodal category and can influence the superior extent of a radiation field. Its incremental effect on systemic treatment may be smaller if high-risk features already justify systemic therapy. Before additional surgery, the multidisciplinary team should specify whether a positive or negative result would change the planned radiation field, systemic strategy or operative objective, balanced against morbidity. A plausible change in management is not itself evidence that routine assessment improves survival.

Alternative injection strategies that increase direct para-aortic mapping are scientifically attractive but have not yet demonstrated sufficient clinical benefit to displace cervical injection. The central research question is not whether fundal or hysteroscopic injection can identify more para-aortic nodes; it is whether the additional nodes provide actionable information that improves outcomes enough to justify a more complex or less reproducible mapping strategy.

## 9. SLN Mapping After Neoadjuvant Therapy and Other Non-Standard Settings

Prospective validation of SLN mapping after neoadjuvant systemic therapy in endometrial cancer remains insufficient. Most accuracy data concern primary staging, and contemporary trial protocols may explicitly exclude neoadjuvant treatment and prior pelvic nodal surgery [50]. Treatment-related changes in flow or viable tumor burden are plausible concerns, rather than mechanisms demonstrated by the primary-staging accuracy studies. The high sensitivity reported in untreated cohorts should therefore not be presented as the expected sensitivity after systemic therapy.

If mapping is considered after neoadjuvant therapy, its limitations should be discussed in a multidisciplinary setting using pretreatment distribution, response imaging, operative findings and the purpose of further nodal information. Fluorescence cannot establish sterilization of an originally involved basin. A prospective study should record pretreatment nodal status, detection by side, reference assessment, response and recurrence; comparison with untreated primary-staging cohorts would require careful adjustment. No routine post-neoadjuvant SLN recommendation is proposed here.

Reoperative surgery is a separate evidence problem. Mapping after supracervical hysterectomy has been described using injection into the retained cervical stump [51]. The 2025 ESGO–ESTRO–ESP guidelines recommend avoiding re-surgery in low-risk patients without residual disease. When completion surgery is indicated and the cervix remains accessible, SLN assessment by cervical injection can be performed; failed mapping is managed according to the principles used in primary surgery. After total hysterectomy, standard cervical injection SLN assessment is no longer feasible. Systematic pelvic lymphadenectomy should be considered only in patients who are not at low risk and when its result could modify adjuvant treatment [9]. Prior nodal dissection and altered anatomy further limit extrapolation from primary-staging accuracy studies. These settings should therefore be distinguished from routine primary staging.

## 10. A Practical Decision Framework

Table 4 summarizes the principal decisions; it is an interpretive framework rather than a prospectively validated decision rule. Table 5 distinguishes prospective diagnostic evidence from guideline recommendations, and Table 6 separates molecular prognostic context from treatment inferences that remain unproven. For each patient, the intended value of additional nodal information should be stated before extending surgery: anatomic staging, radiation-field selection, clarification of suspected extrauterine disease, or a specific change in systemic management.

**Table 5:** Prospective diagnostic evidence and contemporary guidance.

Study or Guidance	Population and Technique	Detection and Accuracy	Endpoint and limitation
FIRES [4]	Clinical stage I; all grades and histologies; cervical ICG. 340 underwent mapping and pelvic reference dissection.	At least one SLN: 86% (293/340). Sensitivity 97.2%; NPV 99.6%; FNR 2.8% among mapped node-positive patients.	Prospective diagnostic cohort; accuracy conditional on successful mapping. Not a survival non-inferiority trial.
SENTI-ENDO [7]	125 eligible early-stage patients; cervical technetium-99m and blue dye.	Patient-level detection 111/125 (88.8%). Sensitivity 84%; NPV 97%; FNR 16% at patient level.	Prospective diagnostic study; contralateral and para-aortic false negatives underline algorithm limitations.
SHREC [5]	257 presumed stage I–II high-risk patients; cervical ICG; pelvic and infrarenal para-aortic reference dissection.	Bilateral 95%. ICG algorithm sensitivity 98%, NPV 99.5%; overall algorithm sensitivity 100%.	Diagnostic endpoint; algorithm definitions affect estimates. No randomized survival comparison.
SENTOR [6]	156 intermediate/high-grade patients; cervical ICG; pelvic reference dissection; para-aortic dissection in 101/126 high-grade patients.	Patient-level 97.4%; bilateral 77.6%. Sensitivity 96%; FNR 4%; NPV 99%.	Prospective diagnostic cohort; subgroup survival equivalence not established.
ESGO–ESTRO–ESP 2025 [9]	Guidance for presumed uterine-confined disease; validated SLN algorithm and ultrastaging.	Detection and FNR: not applicable to a guideline.	Recommendations synthesize evidence; they are not an additional diagnostic or survival trial.

Detection rates have different denominators and should not be pooled or ranked without accounting for population, surgeon experience and algorithm. FNR = false-negative rate; NPV = negative predictive value. FNR is not the proportion of all patients misclassified. FILM [8] compares tracer detection rather than survival and should not be interpreted as an oncologic non-inferiority comparison.

**Table 6:** Molecular context without unvalidated treatment extrapolation.

Subgroup	Interpretation of Nodal Information	What is not Established
POLEmut	Favorable prognosis must be interpreted with anatomic stage and uterine factors. Report nodal burden conventionally.	Omission of staging or routine de-escalation for node-positive disease solely because POLEmut is present.
MMRd	Nodal macrometastasis can add prognostic information; predictive treatment evidence must come from the relevant therapeutic setting.	A subtype-specific adjuvant benefit from treating ITCs or micrometastases.
NSMP	Nodal findings add anatomic information within a heterogeneous group; consider grade and estrogen-receptor status.	A uniform treatment rule across NSMP tumors or validated omission of backup dissection.
p53abn	Adverse biology does not make anatomic staging or radiation-field information redundant.	Routine para-aortic dissection with negative pelvic SLNs, or a specific low-volume treatment rule, solely because p53abn is present.

For every subgroup, report ITCs, micrometastases and macrometastases separately. No prospective study cited here validates a molecular-subtype-specific rule for treating ITCs or micrometastases, nor does a prognostic association establish that treatment benefit differs between subgroups. Any proposed omission of nodal staging or modification of adjuvant therapy on the basis of the interaction between molecular subtype and low-volume disease remains a research question, except where a current guideline explicitly addresses the full clinical scenario. Node-positive POLEmut disease warrants particular caution because early-stage de-escalation evidence cannot simply be extrapolated to stage III disease [9].

## 11. Future Research Priorities

Future research should link technical performance to clinical consequences. Bilateral detection and false-negative rates remain necessary quality metrics, but should be accompanied by treatment changes, lymphatic morbidity, patient-reported outcomes and recurrence or survival endpoints. The SELECT publication [52] describes a prospective observational design in a defined intermediate-risk population; ALICE [48] and SENTIMETREP53 [23] describe randomized designs addressing surgical extent. These protocol publications identify appropriate questions and populations; they do not provide completed oncologic comparisons.

Molecularly annotated cohorts should separately analyze node-negative disease, ITCs, micrometastases and macrometastases, with prespecified adjustment for uterine factors and adjuvant treatment. Prospective rescue studies should define failure by hemipelvis and test when backup dissection changes management. Para-aortic studies should distinguish additional detection from changes in radiation fields or other treatment and from improved outcomes. Laboratory studies, including the SENT-OSNA trial design [50], address another source of variation: how different nodal assessment methods classify small deposits. Dedicated studies are required in post-neoadjuvant and reoperative settings. De-escalation should be tested within explicit clinical/molecular groups, with adequate follow-up and transparent reporting of crossover and additional surgery.

## 12. Conclusions

SLN mapping is now a mature staging strategy in endometrial cancer, and the field has moved beyond the binary comparison with systematic lymphadenectomy. ICG with near-infrared imaging and cervical injection provides the most reproducible bilateral pelvic mapping and should remain the reference technique. Blue dye has lower detection performance, whereas technetium-99m remains a valid but logistically more demanding alternative. Injection-site studies highlight the anatomical trade-off between reliable pelvic mapping and greater para-aortic visualization. Persistent mapping failure should be managed by hemipelvis and baseline staging indication, with particular attention to higher-risk disease and operative feasibility. Prospective studies support diagnostic accuracy in high-risk populations; definitive survival non-inferiority in every histologic or molecular subgroup is not established by these studies. Molecular classification complements nodal staging but does not currently validate subtype-specific treatment rules for ITCs or micrometastases. Selective para-aortic assessment should address a defined management question. Prospective studies must determine whether molecularly integrated nodal strategies change treatment and preserve oncologic outcomes.

## Figures and Tables

**Table 1:** Overlapping coverage in recent reviews and the complementary focus of this article.

Recent Review	Main Emphasis	Key Contribution	Complementary Focus of this Review
Bogani et al., 2024 [11]	General SLN mapping	Evolution, oncologic safety, implementation	Technique determines whether an unmapped side needs rescue staging; molecular status alone does not resolve that decision.
Clark et al., 2024 [12]	Procedure standardization	Injection volume/site, pathology interpretation	Distinguishes improved low-volume detection from proven benefit of treatment escalation.
Leone Roberti Maggiore et al., 2024 [13]	When/how/which patients	Indications and technique	Connects baseline staging indication with side-specific backup dissection and operative feasibility.
Salman et al., 2024 [14]	Contemporary overview	Clinical implementation	Separates selective para-aortic assessment from routine dissection without demonstrated benefit.
Bogani et al., 2025 [16]	Molecular integration	SLN and molecular integration, including low-volume disease and future personalized management	Acknowledges prior molecular integration; subsequent cohorts refine associations without validating subtype-specific surgical or adjuvant rules.
Glaser and Ostby, 2025 [17]	Current practice and surgical staging	SLN implementation and molecular profiling	Compares diagnostic, prognostic and treatment evidence explicitly at unresolved clinical decision points.

## Data Availability

No new data were created or analyzed in this study. Data sharing is not applicable.
